# Association of Rotating Night Shift Work with BMI and Abdominal Obesity among Nurses and Midwives

**DOI:** 10.1371/journal.pone.0133761

**Published:** 2015-07-21

**Authors:** Beata Peplonska, Agnieszka Bukowska, Wojciech Sobala

**Affiliations:** Department of Environmental Epidemiology, Nofer Institute of Occupational Medicine, Lodz, Poland; Università di Milano, ITALY

## Abstract

**Background:**

Mounting epidemiological evidence suggests that night shift work may contribute to the etiology of increased body weight. The present study aimed to examine association between rotating night shift work and body mass index (BMI), and abdominal adiposity respectively among nurses and midwives.

**Methods:**

A cross-sectional study was conducted among 724 female nurses and midwives, aged 40-60 years (354 rotating night shift and 370 daytime workers) in Łódź, Poland, between 2008 and 2011. Information about occupational history and potential confounders was collected during personal interviews. Anthropometric measurements of body weight, height, waist (WC) and hip (HC) circumference were made, and body mass index (BMI), waist to hip ratio (WHR) and waist to height ratio (WHtR) were calculated. GLM regression models and multinomial logit regression models were fitted to explore the association between night shift work and anthropometric parameters, with adjustment for age, body silhouette at age 20, current smoking status, packyears, marital status, and menopausal hormone therapy use.

**Results:**

Cumulative night shift work showed significant associations with BMI, WC, HC and WHtR, with BMI increasing by 0.477 kg/m^2^ per 1000 night duties and by 0.432 kg/m^2^ per 10000 night shift hours, WC increasing respectively by 1.089 cm and 0.99 cm, and HC by 0.72 cm and WHtR by 0.007 cm for both metrics. Both current and cumulative night work was associated with obesity (BMI≥30kg/m^2^), with OR=3.9 (95%CI:1.5-9.9), in women reporting eight or more night shifts per month.

**Conclusion:**

The results of the study support the previously reported relations between night shift work and development of obesity.

## Introduction

In modern societies, night work has been recognized as one of the most prevalent occupational factors, affecting about 15 to 20% of the working population in Europe and North America [1], and has been linked to some chronic diseases including cardiovascular diseases and cancer [2]. In spite of some inconsistencies, several cross-sectional studies report significant associations between night shift work and increased BMI [3–14]. Recently, Van Drongelen et al.[15] have identified eight longitudinal studies that examined relationship between night shift work and body weight gain, and concluded that evidence for a crude association between shift work and body weight increase is strong but becomes insufficient after controlling for confounders. Since van Drongelen’s review only two longitudinal studies on this topic have been published. Further support for the relationship between night shift work and weight gain was found for women but not for men [16,17].

Even though most of the previous findings on relations between night work and obesity were highly suggestive, several methodological limitations need to be considered. In particular, some of these studies [3,5–9,14] relied on self-reported weight and height, which could have resulted in some imprecision in BMI assessment. Moreover, most of the studies [3–7,9–13] used crude information about the system of work performed by the worker at the moment of study, thus the conclusions regarding causal relationships were limited. Apart from that, the researchers typically used BMI for the assessment of body composition. However, BMI does not distinguish between overweight due to muscle or fat tissue. The more accurate measures of abdominal adiposity, which were implicated for example in metabolic disorders and breast cancer, have rarely been considered.

Given the limitations of previous research on the relationship between night shift work and body weight as well as the scarcity of data for abdominal adiposity in relation to night work, we made use of the data obtained from a cross-sectional study on nurses and midwives in order to further explore this topic. In particular, we used information about current and past night shift work and examined its association with BMI and several measures of abdominal adiposity.

## Methods

This cross-sectional study was described before. [18] In brief, nurses and midwives registered at the Local Registry of the Chamber of Nurses and Midwives in Lodz, Poland were eligible if met the inclusion criteria, i.e. the age of 40–60 years and current employment as a nurse or midwife in public health care setting in Lodz. A total of 1117 nurses and midwives (~30% of all registered in this age group) were randomly selected based on the registry database, and out of these, 924 (83%) were contacted. Current employment status was confirmed for 866 women, and 725 women agreed to take part in the study. One of the women refused to have anthropometric measurements made and was excluded.

Nurses and midwives working rotating night shifts typically have fast rotating 12 hours’ long duties between 7 p.m. and 7 a.m. Also typically night shift is usually followed by a day off. According to the legal regulations in Poland, for health care workers, the total duration of the working week comprising five working days, is 37h and 55 min. The working schedules vary depending on the setting, and also on a month to month basis within the same setting. A structured questionnaire was administered during in-person interviews to elicit information on occupational history, demographics, medical and reproductive history, hormone use, physical activity (according to the International Physical Activity Questionnaire (IPAQ)[19], smoking, alcohol use, diet, and sleep quality (according to Pittsburgh Sleep Quality Questionnaire). [20]. Morning or evening preference was determined “lark” or “owl” personality, with the explanation of these terms being provided in the questionnaire. Women were also asked about their current weight and height, and had to indicate their body shape image (current and at age 20) using a 9-level silhouette scale [21].

Anthropometric measurements were performed in the morning hours (6:00–10:00) by trained nurses at least one week after the interview.

The study was approved by the ethical review board at the Nofer Institute of Occupational Medicine. A signed informed consent was obtained from each study participant.

### Anthropometric measures

Anthropometric measurements were performed on average 11 days after the interview. Weight, height, and waist and hip circumferences were measured twice in a standing position and the means for each measurement were used for analysis. (Pearson’s correlation coefficient between each pair of measurements was high (>0.99).

Height and weight were determined using a conventional stadiometer and scales. Respondents were asked to take off their shoes before measurements. The waist circumference was measured at the level of the umbilicus, and the hip circumference at the widest part, over the buttocks. Based on the anthropometric measurements of weight(kg), height(cm), waist(WC)(cm) and hip(HC)(cm) circumferences, the following parameters were calculated: body mass index (BMI), waist to hip ratio (WHR), and waist to height ratio (WHtR).

BMI was categorized, according to the classical WHO definition [22], as normal if between 18.5 kg/m^2^ and 24.99 kg/m^2^, overweight if between 25kg/m^2^ and 29.99kg/m^2^, and obese if ≥30 kg/m^2^. In categorizing WC and WHR, we adopted the following cutoffs: WC of 88 cm and WHR of 0.85. [23]. For hip circumference, we distinguished three categories: HC< 102.5cm, 102.5cm-108.0cm, and >108.0cm (the latter were associated with increased risk of breast cancer in the EPIC study [24]). For WHtR, we used a boundary value of 0.55 for women of less than 50 years of age, and 0.6 for women of 50 or more years of age.[25].

The perceived body shape images (silhouettes) were grouped into four categories following recommended classification [26]: pictures 1–3 represented underweight (BMI≤19.99 kg/m^2^), pictures 4 and 5 normal weight (BMI range of 20–24.99 kg/m^2^), pictures 6 and 7 overweight (BMI range of 25kg/m^2^–29.99 kg/m^2^), and pictures 8 and 9 obesity (BMI>30 kg/m^2^).

### Night shift exposure assessment

Data on current job collected via the questionnaire were used. The current job characteristics and total rotating night shift work history were analyzed. The following variables were considered: current rotating night shift work *(yes/no)*, night shift work frequency *(the number of night duties per month)*, total duration (in years) of jobs with night shift work, *(obtained by summing up the duration of each job with night shifts)*. Additionally, the cumulative number of night shifts was calculated using the duration of each job and the average frequency of night shifts on this job, as well as cumulative night shift hours over the entire work history, based on the start and end hours of the night shift for each job. For the purpose of statistical analysis, night shift work variables were expressed as the following categories: frequency of 2–7, and 8 or more night duties per month; duration of ≤10 yr, >10-≤20 yr and >20 yr; cumulative night shifts of ≤1000, >1000 -≤2000 and >2000; and cumulative hours of night shift work of ≤12000 h, >12000h-≤24000h and >24000 h.

### Statistical analysis

Basic characteristics of the study groups by their current night shift work status were compared with Student’s t-test for the continuous, and chi-square test for the categorical variables.

The list of potential confounders included age (continuous, in years), menopausal status (pre- and postmenopausal), marital status (single, married/cohabitating, divorced/in separation, widow), current smoking (never smoker, past smoker and current smoker), number of cigarettes currently smoked per day (categories: 0, 1–4, 5–14, 15–24, ≥25), smoking years (continuous, in years), and packyears (continuous, in packyears), body shape image at age 20 (continuous, scores 1–9), total physical activity (continuous, in MET*h per week (metabolic equivalent ratio of the metabolic rate to a standard resting metabolic rate of 1 during quiet sitting), recreational physical activity (continuous, in MET*h per week, and in the following categories: yes(if any)/no), number of full-term births (0, 1–2, ≥3), quality of sleep index (PSQI)(continuous, scores 0–21), average duration of habitual sleep (continuous, in hours), chronotype (morning type, evening type), alcohol use (the number of alcoholic drinks per week), current use of oral contraceptives (yes/no), ever use of oral contraceptives (yes/no); current use of menopausal hormone replacement therapy (MHT)(yes/no), and ever use of MHT (yes/no). All the potential confounders listed above were included in the models and backward stepwise analysis was applied to select the ones important for final models. The AIC criterion [27] for selecting variables was used. Age, body shape image at age 20, current smoking status, packyears, marital status and current MHT use were retained in the final models.

In Poland nurses are a relatively homogenous group with respect to the socioeconomic status (SES); they have a similar background education and occupation. Therefore, in the present study SES was not considered as a potential confounder.

To assess the association between night shift work characteristics and anthropometric measures, the GLM regression models were used with inverse Gaussian and identity link fitted with each anthropometric measure as the dependent variables, and night shift work characteristics and a set of covariates described above as the independent variables. Odds ratios with 95% confidence intervals were calculated using the multinomial logit regression models to explore the associations of the night shift work with BMI categories of overweight and obesity, and abdominal obesity as measured with anthropometric parameters.

In addition, we tested the chronotype as a potential modifier and examined its possible interaction with night work variables. In none of the regression analyses, was the chronotype found to be a statistically significant modifier and thus the results are presented for the total population, irrespective of the chronotype.

Statistical analyses were performed using R version 3.1.1 (Vienna, Austria).

## Results

The basic characteristics of the study population according to the current status of night shift work are presented in Table 1. Women currently working on rotating night shifts had on average a longer history of night shift work than did the women currently working only on days (25.4 yr vs. 12.1 yr). In the latter group, as much as 83% had resigned from night shift work more than five years before the present study (data not shown). Crude values of the arithmetic means, with standard deviations, and the frequencies according to the categories of anthropometric measures are displayed in Table 2. Obesity (BMI≥30kg/m^2^) and abdominal obesity as denoted by WHR>0.85 and WHtR>0.55(0.6) were slightly more prevalent among women currently working on rotating night shifts but the differences were not statistically significant.

**Table 1:** Selected characteristics of Polish nurses and midwives. A cross-sectional study.

Characteristics	Rotating night shifts n = 354		Day shifts n = 370		p-diff
	N (%)	mean (SD)	N (%)	mean (SD)	
**Mean age (years)**		48.3 (5.2)		50.2 (5.3)	**<0.001**
**Marital status**					0.586
Single	19 (5)		16 (4)		
Married / Cohabitating	273 (77)		276 (75)		
Divorced / in separation	45 (13)		54 (15)		
Widow	17 (5)		24 (6)		
**Menopausal status**					**<0.001**
Pre-	231 (65)		191 (52)		
Post-	123 (35)		179 (48)		
**Number of full term births**					0.105
0	27 (8)		27 (7)		
1	107 (30)		130 (35)		
2	185 (52)		193 (52)		
3–4	35 (10)		20 (6)		
**Smoking (%)**					**0.014**
Current	123 (35)		97 (26)		
Past	82 (23)		115 (31)		
Non-smoker	149 (42)		158 (43)		
**Smoking years (years)**		15.1 (14.5)		14.4 (14.7)	0.487
**Pack-years**		9.9 (11.3)		8.5 (10.5)	0.096
Total physical activity (MET*hours per week)		242.7 (78.2)		202.6 (87.5)	**<0.001**
**Recreational activity** ^a^					**0.028**
No	111 (31)		88 (24)		
Yes	242 (69)		281 (76)		
**Chronotype** ^a^					0.568
Morning type	182 (51)		197 (54)		
Evening type	172 (49)		171 (46)		
**Quality of sleep (PSQI score)**		6.3 (3.0)		6.8 (3.6)	**0.041**
**Average duration of sleep (hours)**		6.7 (1.1)		6.2 (1.1)	**<0.001**
**Current MHT use (among postmenopausal women)**					0.161
No	341 (96)		347 (94)		
Yes	13 (4)		23 (6)		
**Alcohol consumption (drinks per week)**		0.6 (0.7)		0.6 (0.7)	0.679
**Total duration of night shift work (years)**		25.4 (7.1)		12.1 (8.2)	**<0.001**

^a^ missing information: for recreational activity in one night shift working woman and one working only during the day and for chronotype in two day shifts nurses.

Abbreviations:

MET—metabolic equivalent ratio of the metabolic rate to a standard resting metabolic rate of 1 during quiet sitting

PSQI—Pittsburgh Sleep Quality Index

MHT—menopausal hormone replacement therapy.

**Table 2:** Arithmetic means (SD) of anthropometric measures and prevalence of overweight/obesity by current system of work in the study population.

Anthropometric characteristics	Rotating night shifts n = 354		Day shifts n = 370		p-diff
	N(%)	mean(SD)	N(%)	mean(SD)	
**BMI (kg/m** ^**2**^ **)**		27.1(4.7)		27.0 (4.7)	0.739
Normal weight (BMI<24.99 kg/m^2^)	125 (35)		142 (38)		0.527
Overweight (BMI 25–29.99 kg/m^2^)	141 (40)		148 (40)		
Obesity (BMI≥30.0 kg/m^2^)	88 (25)		80 (22)		
**Waist circumference (WC) (cm)**		83.9 (11.7)		83.8 (12.0)	0.946
WC≤88cm	245 (69)		250 (68)		0.693
WC>88cm	109 (31)		120 (32)		
**Hip circumference (HC) (cm)**		103.9 (9.1)		104.0 (10.3)	0.945
HC<102.5cm	162 (46)		185 (50)		0.463
HC≥102.5-<108cm	70 (20)		63 (17)		
HC≥108cm	122 (34)		122 (33)		
**Waist to hip ratio (WHR)**		0.81 (0.07)		0.80 (0.06)	0.840
WHR≤0.85	262 (74)		281 (76)		0.606
WHR>0.85	92 (26)		89 (24)		
**Waist to height ratio (WHtR)**		0.52 (0.07)		0.52 (0.07)	0.895
WHtR≤0.55^a^	277 (78)		300 (81)		0.352
WHtR>0.55^a^	77 (22)		70 (19)		

^a^cut-off 0.55 for women aged <50; 0.6 for women aged ≥50.

Current night shift work characteristics did not show significant associations with the continuous variables of the anthropometric measures (Table 3). The analysis revealed positive and statistically significant associations for cumulative night shift work exposure expressed as the total number of night shifts (or of night shift hours) and BMI, WC, HC, and WHtR, with BMI increasing by 0.477 kg/m^2^ per each 1000 night shift duties and by 0.432 kg/m^2^ per 10000 night shift hours, WC increasing respectively by 1.089 cm and 0.997 cm, and HC by 0.72 cm, and WHtR 0.007 for both metrics. We also observed a weak positive association between the duration of night shift work and the BMI and WHtR, which was of borderline significance (p = 0.075 and p = 0.073 respectively).

**Table 3:** Association between night shift work characteristics and anthropometric measures in the study population.

		β coeficient^a^(p-value)			
Night shift work (NSW) characteristics	BMI	WC	HC	WHR	WHtR
Current NSW^b^	0.482(0.129)	0.941(0.252)	0.721(0286)	0.003(0.479)	0.007(0.170)
Frequency of NSW^b^	0.082(0.080)	0.166(0.171)	0.123(0.218)	0.001(0.339)	0.001(0.118)
Duration of NSW (per 10 yr)	0.282(0.075)	0.663(0.105)	0.400(0.230)	0.003(0.231)	0.005(0.073)
Cumulative night shifts (per 1000 night shifts)	0.477**(0.007)**	1.089**(0.018)**	0.724(0.053)	0.004(0.094)	0.007**(0.009)**
Cumulative night shift hours (per 10000 h)	0.432**(0.004)**	0.997**(0.010)**	0.716**(0.023)**	0.004(0.095)	0.007**(0.006)**

^a^ adjusted for age (continuous, in years), smoking (never, ex-, current), packyears (continuous), marital status (never married, married/cohabitating, divorced/in separation, widow), body silhouette at age 20 (continuous, in the range of 1–9), and current MHT use (yes/no)

^b^reference—day shift nurses.

Night shift work, both current and cumulative, was consistently associated with obesity (BMI≥30kg/m^2^), with OR of 3.9 (95%CI:1.5–9.9) in women reporting eight or more night shifts per month, and statistically significant positive trends were noted for every parameter of cumulative night shift work (Table 4). A higher frequency of night shifts was also significantly associated with abdominal obesity, with OR = 2.4 (95%CI:1.2–4.5) for WC>88cm, OR = 2.8 (95%CI:1.3–6.0) for HC≥108cm, OR = 2.4 (95%CI:1.2–4.9) for WHR>0.85 and OR = 2.7 (95%CI:1.3–5.6) for WHtR>0.55 (0.6). We also observed significant positive trends for the association between cumulative hours of night shift work and BMI>30kg/m^2^, WC>88 cm, and HC≥102.5cm- 108cm as well as the number of cumulative night shifts and WHtR>0.55(0.6). All the remaining associations were positive and in the expected direction, although not supported with statistically significant tests.

**Table 4:** Adjusted^a^ odds ratios (OR) of overweight/obesity in the study population.

Night shift work (NSW) characteristics (n)	BMI		WC	HC		WHR	WHtR
	25–29.9 kg/m^2^	≥30.0 kg/m^2^	>88cm	≥102.5 -<108cm	≥108cm	>0.85	>0.55^d^
	N = 289	N = 168	N = 229	N = 133	N = 244	N = 181	N = 146
**Current NSW** ^b^ **(354)**	1.2(0.8–1.7)	1.5(1.0–2.3)	1.01(0.8–1.5)	1.4(0.9–2.1)	1.4(0.9–2.0)	1.3(0.9–1.8)	1.3(0.9–2.0)
*P*	*0*.*322*	***0*.*048***	*0*.*689*	*0*.*071*	*0*.*119*	*0*.*182*	0.155
**Frequency of NSW** ^b^							
2-7nights/month (312)	1.2(0.8–1.7)	1.4(0.9–2.1)	1.0(0.7–1.4)	1.5(0.9–2.3)	1.2(0.9–1.8)	1.1(0.8–1.7)	1.2(0.8–1.9)
≥ 8 nights/month (42)	2.0(0.8–4.7)	3.9(1.5–9.9)	2.4(1.2–4.5)	1.6(0.6–4.2)	2.8(1.3–6.0)	2.4(1.2–4.9)	2.7(1.3–5.6)
p-trend^c^	*0*.*203*	***0*.*033***	*0*.*503*	*0*.*077*	*0*.*096*	*0*.*088*	*0*.*139*
**Duration of NSW**							
≤ 10 yr (193)	1.0	1.0	1.0	1.0	1.0	1.0	1.0
>10—≤ 20yr (177)	1.1(0.7–1.7)	1.2(0.7–2.2)	1.2(0.7–1.9)	1.2(0.7–2.1)	1.0(0.6–1.7)	1.3(0.8–2.1)	1.0(0.6–1.8)
>20 yr (354)	1.1(0.8–1.7)	1.5(0.9–2.6)	1.2(0.8–1.7)	1.4(0.9–2.4)	1.3(0.8–1.9)	1.0(0.7–1.6)	1.3(0.8–2.1)
p-trend^c^	*0*.*203*	***0*.*029***	*0*.*327*	*0*.*071*	*0*.*233*	*0*.*459*	*0*.*141*
**Cumulative night shifts**							
≤1000 (211)	1.0	1.0	1.0	1.0	1.0	1.0	1.0
>1000-≤2000 (254)	1.1(0.7–1.7)	1.5(0.9–2.7)	1.3(0.8–2.0)	1.5(0.9–2.4)	1.3(0.8–2.0)	1.1(0.7–1.7)	1.3(0.8–2.2)
>2000 (259)	1.3(0.8–2.0)	2.0(1.2–3.5)	1.3(0.9–2.0)	1.4(0.8–2.3)	1.4(0.9–2.2)	1.3(0.8–2.0)	1.6(0.9–2.5)
p-trend^c^	*0*.*345*	***0*.*002***	*0*.*059*	*0*.*078*	*0*.*128*	*0*.*204*	***0*.*051***
**Cumulative night shift hours**							
≤12000 (228)	1.0	1.0	1.0	1.0	1.0	1.0	1.0
>12000- ≤24000 (248)	1.1(0.7–1.7)	1.6(0.9–2.7)	1.3(0.8–2.0)	1.5(0.9–2.5)	1.3(0.8–2.0)	1.0(0.7–1.6)	1.3(0.8–2.2)
>24000 (248)	1.3(0.9–2.0)	2.1(1.3–3.6)	1.3(0.9–2.0)	1.5(0.9–2.5)	1.5(1.0–2.3)	1.2(0.8–1.9)	1.5(0.9–2.4)
p-trend^c^	*0*.*229*	***0*.*001***	***0*.*045***	***0*.*048***	*0*.*076*	*0*.*191*	***0*.*038***

^a^ adjusted for age (continuous, in years), smoking (never, ex-, current), packyears (continuous), marital status (never married, married/cohabitating, divorced/in separation, widow), body silhouette at age 20 (continuous in the range 1–9), and current MHT use (yes/ no)

^b^reference—day shift nurses

^c^ p trends for continuous variables describing work characteristics

^d^cut-off of 0.55 for women aged <50; 0.6 for women aged ≥50.

### Comparison of the self-reported and measured anthropometry

Self-reported body weight and height were compared with the measured values and both comparisons showed high correlations (Pearson’s correlation coefficient of 0.96 for weight and 0.95 for height). We observed a tendency for underreporting weight, particularly among heavier women. On the other hand, shorter women tended to report a slightly higher body height than respective measurements. The correlation between BMI calculated from the measured and reported values was also high (Pearson’s correlation coefficient 0.95), with some tendency for a lower BMI calculated from the reported weight and height when compared to the measured values. This difference tended to increase with increasing BMI based on measurement results.

We also compared the subjects’ classification according to WHO categories of BMI, using BMI calculated from physical measurements and BMI classes based on self-perceived current body shape image. The correlation between the two categorizations was satisfactory (Pearson’s correlation coefficient 0.81).

## Discussion

This cross-sectional study of nurses and midwives investigated a possible association between rotating night shift work and the anthropometric measures, and the findings provided support for the growing evidence that night shift work is a risk factor for obesity.

In particular, we observed statistically significant linear associations between cumulative night shift work metrics (number of night duties and hours of night work) and all the anthropometric measures examined. Obesity (BMI≥30kg/m^2^) turned out to be significantly associated with such parameters as current status of night shift work, frequency of night duties of ≥8 per month, duration of night shift work longer than 20 years, total number of night duties higher than 2000, and total number of night shift hours higher than 24 000. These associations revealed positive and significant trends. Abdominal obesity was associated with a high frequency of current night duties and cumulative hours of night shift work.

We identified as many as 20 epidemiological studies that examined either cross-sectionally [3–14,28,29] or longitudinally [16,30–34] the association between night work and anthropometric parameters. Most of them (17) showed positive inferences. The associations reported were of moderate strength, with the highest OR = 1.5 found for obesity among healthcare workers in Denmark [14] and OR = 1.9 for overweight among railway service workers in Italy. The increased risk of weight gain or onset of obesity among night shift workers as compared to daytime workers was reported in the majority [16,17,30,32] of longitudinal studies [16,17,30,32,33,35].

Previous studies were focused mostly on BMI. Abdominal obesity was investigated in only a few studies [4,11,28,31], and the results were inconsistent. Two of them reported a positive association between night work and abdominal obesity as measured with WC [4] or WHR [11] but in two other reports regarding WHR, no such links were observed [28,31]. It has been argued that the commonly used Quetelet index (BMI) may not adequately reflect the risk related to obesity since it cannot separate the lean mass from the fat mass, although, both BMI and WC showed strong correlation with ultrasound assessment of the subcutaneous and visceral adiposity [36]. Measures of central obesity have been recommended to complement BMI measurement to identify individuals with increased risk of obesity-related morbidity [23]. Waist circumference, hip circumference and waist-to-hip ratio were typically measured in the studies examining association between abdominal adiposity and breast cancer, and found to be good predictors [37]. It has also been pointed out that the measures quantifying abdominal adiposity, other than the BMI, are better predictors of adverse cardiovascular effects of overweight or obesity [25]. Our study provides evidence that a more intense and prolonged night shift is associated with central obesity. However, given the paucity of previous epidemiological data, further research is warranted to confirm our findings.

Previous research used mostly crude information about night work status and categorized workers into simply night shift or day shift workers. A more detailed characteristics of intensity of night shift work, such as the frequency of night duties was considered in only one analysis, namely Nurses’ Health Study II in US (NHS II)[8]. This study revealed an increase in odds ratio for obesity with an increasing frequency of night duties, which is consistent with our own findings. Two studies [5,6] examined the duration, but no other cumulative metrics, of night work, and both reported positive relationship between night work duration and overweight. The total duration of work, often used as an indicator of total exposure, may to some extent misclassify exposure in the case of night shift work. This may occur since the work duration itself does not account for the intensity of night work, which may substantially differ between subjects, depending on the number of night duties within a given period of time (for example 2 vs. 8 night duties per month). As we had obtained detailed data for each job in the subject’s work history, we were able to construct more refined measures of cumulative exposure than simply the exposure duration. We made use of cumulative night shifts and cumulative night shift hours, and these metrics showed much stronger effects than the exposure duration alone.

We also performed a secondary analysis that examined the correlation between self-reported and measured body weight and height. As far as we know, no previous study regarding night shift work and weight-related parameters has described this kind of analysis. Worthy of note is the fact that almost half of the studies addressing the effects of night shift work have relied on self-reported anthropometry [3,5–9,14,17,29]. The high correlation between the data on weight and height from the questionnaire and the actual measurement (Pearson’s correlation coefficient 0.95–0.96 for height and weight, respectively) that we could observe in our study confirmed the accuracy of the subjects’ responses. However, we noted a general tendency among heavier women to report a lower weight, which is consistent with the findings by other authors [38]. When we run an analysis with BMI calculated based on the reported weight and height, the results for the associations with night work characteristics were very similar to those based on BMI calculated from the measurements. This implies that no substantial misclassification would have occurred if we had data only from the questionnaire, and provides some support for the validity of previous studies that relied on self-reported information. However, one should note that we studied the population of nurses and midwives, which is in general better educated than the blue collar workers usually working on night shifts. Thus our findings may not be directly applicable to other populations.

The mechanism linking night shift work with weight gain and the development of obesity has not been fully explained. Nevertheless, such factors as unhealthy dietary habits, low recreational physical activity, sleep deprivation, and disruption of the circadian rhythm have been proposed as the potential causes. Lowden et al. in their literature review identified several unhealthy features of the eating behaviour that affect night workers, these including meal irregularity, higher animal fat, carbohydrate and protein intake coupled with lower dietary fibre consumption, and frequent snacks taken during the night shift [39]. A few studies reported higher total energy consumption [40] or later time of the last meal [9,29] among night shift workers compared to daytime workers. In our study, we did not control for the dietary factors; therefore, the altered eating behaviours may potentially underlie the associations with the night shift work that we observed.

Night shift workers were found to be less involved in sports or recreational physical activity, which was demonstrated in several investigations [9,29,41–44]. We tested physical activity as a potential confounder, using information about the total physical activity, work-related PA domain or leisure-time PA domain. All of these variables turned out to be insignificant covariates when other important characteristics were introduced in the multivariate analyses. A higher energy expenditure in workers during a night duty, which results from staying awake at night [45] may not counterbalance the excessive energy intake in this group.

Sleep deprivation and circadian rhythm disruption are the remaining potential causes to be considered. As postulated by Garaulet et al., the disruption of the circadian rhythm and the lack of sleep at night may affect the processes related to metabolism and the feeling of hunger, which may excess energy intake, especially in the evening time. [46]. It has been demonstrated that the short duration of sleep decreases leptin and increases ghrelin concentration, which is likely to increase appetite and weight gain [47]. Furthermore, neuroanatomical interactions between the suprachiasmatic nuclei (biological clock) and paraventricular nucleus (involved in the regulation of appetite) of the hypothalamus have been described [48].

The strength of our study is that it was conducted in a well-characterized population of nurses and midwives who due to their background education represent a reliable population, with usually higher participation rates and better recall than in the general population. The response rate was relatively high. The data were collected via a face-to-face interview by trained interviewers, and detailed information on both current work characteristics and lifetime occupational history was obtained. Anthropometric measurements were performed by well-trained personnel. All the major confounding factors were evaluated, including body shape at age 20. The latter one allowed us to control for BMI before the subject had started to work.

There are also some limitations of the present study. One of them is the recall bias which is typical for most questionnaire-based studies and which could affect information about past exposure, such as for example the intensity of night work in early periods of employment. This could have resulted in some misclassification of exposure, which although non differential, may have caused some attenuation of the associations we recorded. Besides, the cross-sectional design limited drawing conclusions about the causal inferences. In order to address this limitation, we used information not only about the current but also lifetime history of night shift work.

The “healthy survival effect” may have also had some effects on the results of analysis by the current system of work, given that the day workers had performed some night work in the past. If the reason for quitting night work was poor health and obesity, then the results of the cross-sectional analysis by the current system of work could be affected with some bias towards the null. We were not able to control for this phenomenon since we did not ask the respondents about the reasons for changing employment and we did not have the anthropometric measurements from the past.

A potential limitation of our study is also related to the lack of information about such factors as stress or work family balance. Accordingly, some residual confounding may be present. Nevertheless, in some previous reports no difference in the level of stress has been observed between nurses working on rotating night shifts and those working only during the days.[49] Moreover, the research addressing relations between work-family balance and obesity has been sparse and inconsistent [50].

Another potential limitation is associated with the cut-off points that we adopted to determine obesity. In our study, BMI was categorized into normal weight, overweight and obesity according to the WHO-recommended standard [22] that has been commonly used by other researchers. Since no normal values or standards that could be generalized to every population have been formulated for all the other anthropometric measures, determining the cut-offs for these measures was more problematic. For WC and WHR, we adopted the cut-off points from a WHO report [23] regarding assessment of the metabolic syndrome or “metabolic complications” associated with obesity in Caucasians [51]. However, the same WHO report indicated that these cut-off points differed between populations, and thus there have been no clear WHO recommendations to use them for the risk assessment [23]. For the categorization of the HC values, we used data from the EPIC study [24] investigating relationships between the risk of breast cancer and body size. In this study, the HC categories of 102.5–107.9 cm and ≥108 cm were two upper quintiles in a large cohort of 176 886 women, aged 18–80 yr, coming from nine European countries. Nevertheless, due to the wide age range and the lack of representation of the Central and Eastern European countries, these categories may not be applicable to the Polish population. It is worth mentioning that in our study as much as 52% women had the hip circumference ≥102.5 cm, which is roughly 12% more when compared to the EPIC cohort. For WHtR categorization, we adopted the value of 0.55 for age of less than 50 yr and 0.6 for ages of 50 yr or more, following the recommendations by Schneider et al. based on two large German cohort studies of cardiovascular events and mortality [25]. Previously, a general boundary value of 0.5 for WHtR was recommended [52]. The application of this cut-off in our population would imply that more than 50% of the women studied are at an increased risk of health outcome related to obesity.

In conclusion, the results of our study support the previously reported relationship between night shift work and the development of obesity, and provide evidence that night work may be associated with central adiposity. Although future prospective studies are needed to confirm these findings, the observed inferences imply that a more intense and prolonged night shift work may predispose to overweight and to central obesity in particular. Further studies are also warranted to better understand the mechanism underlying weight gain among night shift workers.

## References

[1] Straif K, Baan R, Grosse Y, Secretan B, El GF, Bouvard V, et al Carcinogenicity of shift-work, painting, and fire-fighting. Lancet Oncol 2007; 8: 1065–1066. 19271347 10.1016/S1470-2045(07)70373-X

[2] Wang XS, Armstrong ME, Cairns BJ, Key TJ, Travis RC. Shift work and chronic disease: the epidemiological evidence. Occup Med (Lond) 2011; 61: 78–89. 21355031 10.1093/occmed/kqr001 PMC3045028

[3] Zhao I, Bogossian F, Song S, Turner C. The association between shift work and unhealthy weight: a cross-sectional analysis from the Nurses and Midwives' e-cohort Study. J Occup Environ Med 2011; 53: 153–158. 21270661 10.1097/JOM.0b013e318205e1e8

[4] Macagnan J, Pattussi MP, Canuto R, Henn RL, Fassa AG, Olinto MT.Impact of nightshift work on overweight and abdominal obesity among workers of a poultry processing plant in southern Brazil. Chronobiol Int 2012; 29: 336–343. 10.3109/07420528.2011.653851 22390246

[5] Marqueze E, Lemos L, Soares N, Lorenzi-Filho G, Moreno C. Weight gain in relation to night work among nurses. Work 2012; 41: 2043–2048. 10.3233/WOR-2012-0429-2043 22317017

[6] Kim MJ, Son KH, Park HY, Choi DJ, Yoon CH, Lee HY, et al Association between shift work and obesity among female nurses: Korean Nurses' Survey. BMC Public Health 2013; 13: 1204 10.1186/1471-2458-13-1204 24354395 PMC3878177

[7] Smith P, Fritschi L, Reid A, Mustard C. The relationship between shift work and body mass index among Canadian nurses. Appl Nurs Res 2013; 26: 24–31. 10.1016/j.apnr.2012.10.001 23158849

[8] Ramin C, Devore EE, Wang W, Pierre-Paul J, Wegrzyn LR, Schernhammer ES. Night shift work at specific age ranges and chronic disease risk factors. Occup Environ Med 2015; 72:100–107. 10.1136/oemed-2014-102292 25261528 PMC4289641

[9] Geliebter A, Gluck ME, Tanowitz M, Aronoff NJ, Zammit GK. Work-shift period and weight change. Nutrition 2000; 16: 27–29. 10674231 10.1016/s0899-9007(99)00228-2

[10] Barbadoro P, Santarelli L, Croce N, Bracci M, Vincitorio D, Prospero E, et al Rotating shift-work as an independent risk factor for overweight Italian workers: a cross-sectional study. PLoS One 2013; 8: e63289 10.1371/journal.pone.0063289 23675472 PMC3651162

[11] Sookoian S, Gemma C, Fernandez GT, Burgueno A, Alvarez A, Alvarez A, et al Effects of rotating shift work on biomarkers of metabolic syndrome and inflammation. J Intern Med 2007; 261: 285–292. 17305651 10.1111/j.1365-2796.2007.01766.x

[12] Biggi N, Consonni D, Galluzzo V, Sogliani M, Costa G. Metabolic syndrome in permanent night workers. Chronobiol Int 2008; 25: 443–454. 10.1080/07420520802114193 18484373

[13] Manenschijn L, van Kruysbergen RG, de Jong FH, Koper JW, van Rossum EF. Shift work at young age is associated with elevated long-term cortisol levels and body mass index. J Clin Endocrinol Metab 2011; 96: 1862–1865. 10.1210/jc.2011-1551 21880805

[14] Poulsen K, Cleal B, Clausen T, Andersen LL. Work, diabetes and obesity: a seven year follow-up study among Danish health care workers. PLoS One 2014; 9: e103425 10.1371/journal.pone.0103425 25068830 PMC4113351

[15] van DA, Boot C, Merkus S, Smid T, van der Beek A. The effects of shift work on body weight change—a systematic review of longitudinal studies. Scand J Work Environ Health. 2011; 37:263–275. 10.5271/sjweh.3143 21243319

[16] Kubo T, Oyama I, Nakamura T, Shirane K, Otsuka H, Kunimoto M, et al Retrospective cohort study of the risk of obesity among shift workers: findings from the Industry-based Shift Workers' Health study, Japan. Occup Environ Med 2011; 68: 327–331. 10.1136/oem.2009.054445 20884794

[17] Roos E, Lallukka T, Rahkonen O, Lahelma E, Laaksonen M. Working conditions and major weight gain-a prospective cohort study. Arch Environ Occup Health 2013; 68: 166–172. 10.1080/19338244.2012.686931 23566324

[18] Peplonska B, Bukowska A, Gromadzinska J, Sobala W, Reszka E, Lie JA, et al Night shift work characteristics and 6-sulfatoxymelatonin (MT6s) in rotating night shift nurses and midwives. Occup Environ Med 2012; 69: 339–346. 10.1136/oemed-2011-100273 22368032

[19] Craig CL, Marshall AL, Sjostrom M, Bauman AE, Booth ML, Ainsworth BE, et al International physical activity questionnaire: 12-country reliability and validity. Med Sci Sports Exerc 2003; 35: 1381–1395. 12900694 10.1249/01.MSS.0000078924.61453.FB

[20] Buysse DJ, Reynolds CF III, Monk TH, Berman SR, Kupfer DJ. The Pittsburgh Sleep Quality Index: a new instrument for psychiatric practice and research. Psychiatry Res 1989; 28: 193–213. 2748771 10.1016/0165-1781(89)90047-4

[21] Stunkard AJ, Sorenson T, Schulsinger F. Use of Danish Adoption Register for the study of obesity and thinness. in The genetics of neurological and psychiatric disorders. 1983; 115–120. 6823524

[22] WHO. Physical status: the use and interpretation of anthropometry: report of a WHO expert committe. WHO Technical Report Series 1995; 854: 1–452. 8594834

[23] WHO 1. Waist circumference and waist-hip ratio. Report of a WHO Expert Consultation. 2008; 1–39.

[24] Lahmann PH, Hoffmann K, Allen N, van Gils CH, Khaw KT, Tehard B, et al Body size and breast cancer risk: findings from the European Prospective Investigation into Cancer And Nutrition (EPIC). Int J Cancer 2004; 111: 762–771. 15252848 10.1002/ijc.20315

[25] Schneider HJ, Friedrich N, Klotsche J, Pieper L, Nauck M, John U, et al The predictive value of different measures of obesity for incident cardiovascular events and mortality. J Clin Endocrinol Metab 2010; 95: 1777–1785. 10.1210/jc.2009-1584 20130075

[26] McElhone S, Kearney JM, Giachetti I, Zunft HJ, Martinez JA. Body image perception in relation to recent weight changes and strategies for weight loss in a nationally representative sample in the European Union. Public Health Nutr 1999; 2: 143–151. 10933634 10.1017/s1368980099000191

[27] Kadane JB, Lazar NA. Methods and criteria for model selection. Journal of the American Statistical Association 2004; 99: 279–290.

[28] Esquirol Y, Bongard V, Mabile L, Jonnier B, Soulat JM, Perret B. Shift work and metabolic syndrome: respective impacts of job strain, physical activity, and dietary rhythms. Chronobiol Int 2009; 26: 544–559. 10.1080/07420520902821176 19360495

[29] Peplonska B, Burdelak W, Krysicka J, Bukowska A, Marcinkiewicz A, Sobala W, et al Night shift work and modifiable lifestyle factors. Int J Occup Med Environ Health 2014; 27: 693–706. 10.2478/s13382-014-0298-0 25218108

[30] Niedhammer I, Lert F, Marne MJ. Prevalence of overweight and weight gain in relation to night work in a nurses' cohort. Int J Obes Relat Metab Disord 1996; 20: 625–633. 8817356

[31] van Amelsvoort LG, Schouten EG, Kok FJ. Duration of shiftwork related to body mass index and waist to hip ratio. Int J Obes Relat Metab Disord 1999; 23: 973–978. 10490804 10.1038/sj.ijo.0801028

[32] Suwazono Y, Dochi M, Sakata K, Okubo Y, Oishi M, Tanaka K, et al A longitudinal study on the effect of shift work on weight gain in male Japanese workers. Obesity (Silver Spring) 2008; 16: 1887–1893. 18535539 10.1038/oby.2008.298

[33] Watari M, Uetani M, Suwazono Y, Kobayashi E, Kinouchi N, Nogawa K. A longitudinal study of the influence of smoking on the onset of obesity at a telecommunications company in Japan. Prev Med 2006; 43: 107–112. 16750561 10.1016/j.ypmed.2006.04.012

[34] Bolt HM, Roos PH, Thier R. The cytochrome P-450 isoenzyme CYP2E1 in the biological processing of industrial chemicals: consequences for occupational and environmental medicine. Int Arch Occup Environ Health 2003; 76: 174–185. 12690492 10.1007/s00420-002-0407-4

[35] van Amelsvoort LG, Schouten EG, Kok FJ. Duration of shiftwork related to body mass index and waist to hip ratio. Int J Obes Relat Metab Disord 1999; 23: 973–978. 10490804 10.1038/sj.ijo.0801028

[36] Borruel S, Molto JF, Alpanes M, Fernandez-Duran E, varez-Blasco F, Luque-Ramirez M, et al Surrogate markers of visceral adiposity in young adults: waist circumference and body mass index are more accurate than waist hip ratio, model of adipose distribution and visceral adiposity index. PLoS One 2014; 9: e114112 10.1371/journal.pone.0114112 25479351 PMC4257592

[37] Phipps AM. Handbook of anthropometry: Physical Measures of Human Form in Health and Disease. 2012; 1703–1723.

[38] Elgar FJ, Stewart JM. Validity of self-report screening for overweight and obesity. Evidence from the Canadian Community Health Survey. Can J Public Health 2008; 99: 423–427. 19009930 10.1007/BF03405254 PMC6975656

[39] Lowden A, Moreno C, Holmback U, Lennernas M, Tucker P. Eating and shift work—effects on habits, metabolism and performance. Scand J Work Environ Health 2010; 36: 150–162. 20143038 10.5271/sjweh.2898

[40] Morikawa Y, Miura K, Sasaki S, Yoshita K, Yoneyama S, Sakurai M, et al Evaluation of the effects of shift work on nutrient intake: a cross-sectional study. J Occup Health 2008; 50: 270–278. 18408349 10.1539/joh.l7116

[41] Bushnell PT, Colombi A, Caruso CC, Tak S. Work schedules and health behavior outcomes at a large manufacturer. Ind Health 2010; 48: 395–405. 20720331 10.2486/indhealth.mssw-03

[42] Nakamura K, Shimai S, Kikuchi S, Tominaga K, Takahashi H, Tanaka M, et al Shift work and risk factors for coronary heart disease in Japanese blue-collar workers: serum lipids and anthropometric characteristics. Occup Med (Lond) 1997; 47: 142–146. 9156468 10.1093/occmed/47.3.142

[43] van Amelsvoort LG, Schouten EG, Kok FJ. Impact of one year of shift work on cardiovascular disease risk factors. J Occup Environ Med 2004; 46: 699–706. 15247809 10.1097/01.jom.0000131794.83723.45

[44] Peplonska B, Bukowska A, Sobala W. Rotating night shift work and physical activity of nurses and midwives in the cross-sectional study in Lodz, Poland. Chronobiol Int 2014; 31: 1152–1159. 10.3109/07420528.2014.957296 25216072

[45] Wehrens SM, Hampton SM, Finn RE, Skene DJ. Effect of total sleep deprivation on postprandial metabolic and insulin responses in shift workers and non-shift workers. J Endocrinol 2010; 206: 205–215. 10.1677/JOE-10-0077 20479040

[46] Garaulet M, Ordovas JM, Madrid JA. The chronobiology, etiology and pathophysiology of obesity. Int J Obes (Lond) 2010; 34: 1667–1683. 20567242 10.1038/ijo.2010.118 PMC4428912

[47] Taheri S, Lin L, Austin D, Young T, Mignot E. Short sleep duration is associated with reduced leptin, elevated ghrelin, and increased body mass index. PLoS Med 2004; 1: e62 15602591 10.1371/journal.pmed.0010062 PMC535701

[48] Kalra SP, Dube MG, Pu S, Xu B, Horvath TL, Kalra PS. Interacting appetite-regulating pathways in the hypothalamic regulation of body weight. Endocr Rev 1999; 20: 68–100. 10047974 10.1210/edrv.20.1.0357

[49] Barton J, Spelten E, Totterdell P, Smith L, Folkard S. Is there an optimum number of night shifts? Relationship between sleep, health and well-being. Work Stress 1995; 9: 109–123. 11539389 10.1080/02678379508256545

[50] Allen TD, Armstrong J. Further examination of the link between work-family conflict and physical health—The role of health-related behaviors. American Behavioral Scientist 2006; 49: 1204–1221.

[51] Han TS, van Leer EM, Seidell JC, Lean ME. Waist circumference action levels in the identification of cardiovascular risk factors: prevalence study in a random sample. BMJ 1995; 311: 1401–1405. 8520275 10.1136/bmj.311.7017.1401 PMC2544423

[52] Ashwell M, Hsieh SD. Six reasons why the waist-to-height ratio is a rapid and effective global indicator for health risks of obesity and how its use could simplify the international public health message on obesity. Int J Food Sci Nutr 2005; 56: 303–307. 16236591 10.1080/09637480500195066

